# Hand hygiene improvement in Nigeria: challenges and opportunities

**DOI:** 10.1186/2047-2994-4-S1-P145

**Published:** 2015-06-16

**Authors:** S Kama-Kieghe, B Okeke

**Affiliations:** 1 General Practitioner, NHS, UK; 2 Regulatory Affairs, GOJO Industries-Europe Ltd, Milton Keynes, UK

## Introduction

Preventable infections contribute significantly to disease burden in Nigeria. Improving hand hygiene in healthcare, communities and general population can break the chain in the spread of most germs that threaten health and drag on socioeconomic development. Hand hygiene is fundamental to patient safety and important to occupational health – both are interconnected. The occupational health benefits of hand hygiene to healthcare workers provide additional incentives / motivation – acting in their own self-interest as well as duty of care. Implementing hand hygiene improvement at national and facility level involves managing behaviour and expectations and managing change.

## Objectives

This presentation seeks to share our experience in implementing hand hygiene partner programme (HHPP) in Nigeria between 2012 and 2013 with the support of a reputable external private sector partner.

## Methods

The HHPP was implemented in partnership with the Federal Ministry of Health in three key components - stakeholders training, mini targeted local seminars and conferences, carefully structured and committed introduction of WHO multimodal hand hygiene improvement strategy focusing on maternal and child units.

## Results

High level of interest and awareness in hand hygiene in general and the use and role of alcohol based hand rub in infection control were created among key decision makers and opinion leaders, government officials and healthcare workers in Nigeria.

## Conclusion

As we continue to work hard and drive our hand hygiene programme, we hope that the lessons from our experience would be useful in sustaining hand hygiene improvement and public health in Nigeria as well as prove transferable across the big diverse population of over 170 million people and other developing countries as part of strengthening health systems. The Ebola Virus Disease pandemic provides additional advocacy to strengthen infection prevention and control systems. Improvement in hand hygiene is one of the pillars for success.

## Disclosure of interest

S. Kama-Kieghe Employee of: SKD Productivity Center, Shareholder of: Managing Partner, B. Okeke Employee of: GOJO industries-Europe Ltd.

